# Continuous antioxidant drug exposure: a bridge from ideal world to real world of therapy for amyotrophic lateral sclerosis

**DOI:** 10.1093/lifemedi/lnac042

**Published:** 2022-10-08

**Authors:** Jun Wang, Xin-Fu Zhou, Yan-Jiang Wang

**Affiliations:** Department of Neurology and Centre for Clinical Neuroscience, Daping Hospital, Third Military Medical University, Chongqing 400042, China; Chongqing Key Laboratory of Ageing and Brain Diseases, Chongqing 400042, China; Health and Biomedical Innovation, Clinical and Health Sciences, University of South Australia, Adelaide, SA 5001, Australia; Department of Neurology and Centre for Clinical Neuroscience, Daping Hospital, Third Military Medical University, Chongqing 400042, China; Chongqing Key Laboratory of Ageing and Brain Diseases, Chongqing 400042, China

Therapeutic benefits in randomized controlled trials (RCTs) of antioxidant drug edaravone for amyotrophic lateral sclerosis (ALS) were not confirmed in a recent real-world study, with limited time of drug exposure a probable reason. Continuous drug exposure is essential to achieve adequate therapeutic effects to disclose benefits masked by confounders in the real world.

ALS is a progressive and fatal neurodegenerative disease affecting motor neurons, causing muscle weakness, and atrophy, with a median survival of 2–4 years from symptom onset. The worldwide prevalence of ALS is 4.5 per 100,000 people, with an incidence of 1.5–2.4 per 100,000 person-years. However, effective disease-modifying therapies are lacking. Riluzole, a glutamate modulator, is the only drug approved by all major drug authorities, with a limited survival benefit of ~3–5 months. There is an urgent need to develop effective therapies for ALS.

The lack of disease-modifying therapies is partly due to the limited understanding of the pathophysiology of ALS. *SOD1,* encodes the most powerful cellular antioxidant superoxide dismutase (SOD), is the first gene identified in familial ALS patients. The expression of the mutant *SOD1* gene in transgenic animal models mimics the pathophysiology of ALS, suggesting that oxidative stress is essential for the development of ALS. Oxidative damage to proteins, lipids, and DNA is increased in the brain and spinal cord of both familial and sporadic ALS patients [1]. Further supporting evidence comes from epidemiological studies. Intake of antioxidants (vitamin E, polyunsaturated fatty acids, etc.) reduces ALS risk, whereas exposure to lead or pesticides, which activate the production of oxygen radicals and deplete antioxidants in the body, increases the disease risk. These findings support the critical role of oxidative stress in the pathogenesis of ALS. Therefore, oxidative stress is considered a potential therapeutic target for ALS.

Edaravone intravenous injection, initially indicated for acute ischemic stroke, was repurposed to treat ALS due to its antioxidant stress properties. In a phase II clinical trial, short-term (24 weeks) intravenous infusion of edaravone slowed the progression of motor dysfunction, as reflected by a decrease in Revised ALS Functional Rating Scale scores in ALS patients. A confirmatory phase III RCT (MCI186-ALS16) only observed positive responses to edaravone in subpopulations at the early stage of ALS [2]. Based on this finding, a subsequent RCT phase III trial (MCI186-ALS19) focusing on patients with early ALS was performed, and 24-week use of edaravone slowed the progression of ALS by 30% [3]. The extension studies of the above two phase III trials showed that prolonged use (48 weeks) of edaravone benefited motor function in early ALS patients. On this basis, edaravone was approved as the second disease-modifying drug for ALS by the US Food and Drug Administration (FDA) in 2017.

A recent study re-evaluated the safety and effectiveness of edaravone for ALS in real-world cohorts at both early and advanced stages from the German Motor Neuron Disease Network [4]. With a median treatment of ~1 year, patients receiving edaravone did not differ from those receiving standard therapy in terms of the speed of disease progression, survival probability, and time to ventilation in total participants or subpopulations who were at early stage of ALS.

Why were the benefits of edaravone observed in the ideal world not reproduced in the real world? One important explanation is that the treatment benefit of edaravone is mild so that it could be easily compromised by multidudinous confounders in the real world. Although some critical characteristics (such as the age of onset, disease duration, and baseline function score) were matched between different treatment groups, genetics, riluzole standard treatment, comorbidities, and other unknown confounders were not considered, which might counterbalance the therapeutic effects of edaravone.

A critical factor restricting the treatment benefit of edaravone is the limited drug exposure time. The edaravone that is currently available is a liquid formulation. In all previous trials, a single dose of 60 mg/d edaravone was administered intravenously in an alternating cycle of 10 out of 14 days of treatment followed by 14 days off (Fig. 1A). The pharmacokinetics of edaravone show a short elimination half-life of 2.5–3 h after intravenous injection, and the edaravone concentration in blood was extremely low, even undetectable, ~8 h post injection (Fig. 1B). Hence, ALS patients receiving this infusion regimen had inadequate blood concentrations of edaravone for more than half a day per day in the 10-day treatment period and had no edaravone exposure for 18 days out of the 28-day cycle. This means that patients were not exposed to adequate or any edaravone >80% of the time per treatment cycle. Unlike acute ischemic stroke, ALS is a chronic and progressive disease, in which oxidative stress is persistent and gradually aggravates. Long-term and continuous drug exposure is essential to achieve therapeutic effect to disclose benefits masked by confounders in the real world. Moreover, as the changes in oxidative stress during edaravone treatment were not measured in previous RCTs or the recent real world study, it remains unknown whether oxidative stress in the brains of ALS patients was well controlled under this edaravone infusion regimen, although a marker of oxidative stress, 3-nitrotyrosine, was substantially lowered in CSF in the phase 2 study. Therefore, the limitation of the infusion formulation of edaravone makes the recent trials insufficient to test the efficacy of edaravone in ALS, and whether oxidative stress is a valid therapeutic target for ALS.

**Figure 1.**
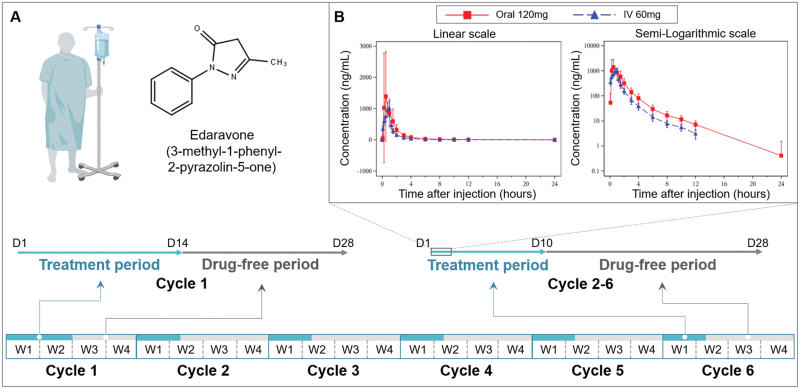
The infusion regimen of edaravone intravenous injection in clinic trials for ALS. (A) Chemical structure of edaravone (3-methyl-1-phenyl-2-pyrazolin-5-one) and the infusion regimen of edaravone intravenous injection in clinic trials for ALS. (B) The pharmacokinetics of edaravone intravenous injection and solid dispersion tablet (From clinical study report of trial NCT04370431).IV, intravenous injection.

Given that long-term and high-frequency infusions could cause infusion-related adverse events and are inconvenient with poor compliance, oral formulations are suitable for multiple doses to maintain drug exposure for ALS. Several oral edaravone formulations have been recently developed with good bioavailability. Oral edaravone suspension (Radicava ORS) has acquired FDA approval recently. The recommended dosage of Radicava ORS is 105 mg once daily with similar pharmacokinetics (an equivalent area under the concentration-time curve and equivalent or higher maximum concentration) as intravenous preparation. The administration regimen is also the same as that of intravenous preparation. Therefore, the sufficient drug exposure time is around 20% of the time per treatment cycle. Whether multiple and continuous administration of oral edaravone has greater benefit in ALS treatment needs to be answered. A solid dispersion tablet of edaravone developed by our group has also showed good bioavailability in phase 1 clinical study (Fig. 1B). In addition, as the half-life of these formulations is still short (2.8–3.9 h), long-acting preparation, or sustained release tablets of edaravone should be developed to improve patient compliance. The development of novel oral edaravone formulations may also provide an opportunity for trials in other chronic neurodegenerative diseases, such as Alzheimer’s disease, which was found to be improved by edaravone in an animal model [5].

Several other antioxidant stress drugs have also shown promising results in clinical trials. Sodium phenylbutyrate-taurursodiol (PB-TURSO) is designed to reduce neuronal death by simultaneously mitigating endoplasmic reticulum stress and mitochondrial dysfunction. Twenty-four-week (twice daily) use of PB-TURSO significantly slowed function decline by 25% in the phase 2 CENTAUR trial, and showed a survival benefit of ~18 months in an open-label extension phase. Recently, Health Canada approved it with conditions for ALS. The phase 3 PHOENIX trial to evaluate the efficacy and safety of 48-week use of PB-TURSO in ALS is now in recruiting. Verdiperstat, another antioxidant stress drug, has been granted fast-track and orphan-drug designations by the FDA and the European Medicine Agency for multiple system atrophy. The phase 2/3 trial to evaluate the efficacy and safety of verdiperstat in ALS is ongoing. The results of these trials are anticipated.

In conclusion, the lack of a treatment benefit of edaravone in the real world provides conceptual lessons for treatment regimens, study designs, and drug development in ALS therapy. Continuous antioxidant drug exposure might be a bridge connecting the gap between the ideal world and the real world. A long-acting oral formulation of edaravone is needed to achieve continuous drug exposure to answer whether edaravone is effective in treating ALS, as well as other neurodegenerative diseases.

## References

[1] Ferrante RJ, Browne SE, Shinobu LA, et al. Evidence of increased oxidative damage in both sporadic and familial amyotrophic lateral sclerosis. J Neurochem. 1997;69:2064–74. 9349552 10.1046/j.1471-4159.1997.69052064.x

[2] Abe K, Itoyama Y, Sobue G, et al. Confirmatory double-blind, parallel-group, placebo-controlled study of efficacy and safety of edaravone (MCI-186) in amyotrophic lateral sclerosis patients. Amyotroph Lateral Scler Frontotemporal Degener. 2014;15:610–7. 25286015 10.3109/21678421.2014.959024 PMC4266079

[3] Writing G, Edaravone ALSSG. Safety and efficacy of edaravone in well defined patients with amyotrophic lateral sclerosis: a randomised, double-blind, placebo-controlled trial. Lancet Neurol. 2017;16:505–12. 28522181 10.1016/S1474-4422(17)30115-1

[4] Witzel S, Maier A, Steinbach R, et al. Safety and effectiveness of long-term intravenous administration of edaravone for treatment of patients with amyotrophic lateral sclerosis. JAMA Neurol. 2022;79:121–30. 35006266 10.1001/jamaneurol.2021.4893 PMC8749709

[5] Jiao SS, Yao XQ, Liu YH, et al. Edaravone alleviates Alzheimer’s disease-type pathologies and cognitive deficits. Proc Natl Acad Sci USA. 2015;112:5225–30. 25847999 10.1073/pnas.1422998112 PMC4413288

